# Primary intraocular lymphoma in a patient with systemic lupus erythematosus

**DOI:** 10.1007/s00277-012-1479-1

**Published:** 2012-05-03

**Authors:** Feng Jung S. H. Woei-A-Jin, Sabina Kersting, Jan Geert Bollemeijer

**Affiliations:** 1 Department of Hematology, Leiden University Medical Center, Albinusdreef 2, 2333 ZA Leiden, The Netherlands; 2 Department of Ophthalmology, Leiden University Medical Center, Albinusdreef 2, 2333 ZA Leiden, The Netherlands

Dear Editor,

Establishment of an intraocular lymphoma may form a diagnostic challenge, especially when biopsy is contraindicated due to the anatomic site of the lesion. In recent years, vitreous IL-10 levels above 400 pg/mL (sensitivity 80 %, specificity 99 %) and a vitreous IL-10 to IL-6 ratio greater than 1.0 (sensitivity 74 %, specificity 75 %) were found to be associated with the presence of an intraocular lymphoma [1, 2]. We present a case where retinal biopsy was not possible.

**Fig. 1:**
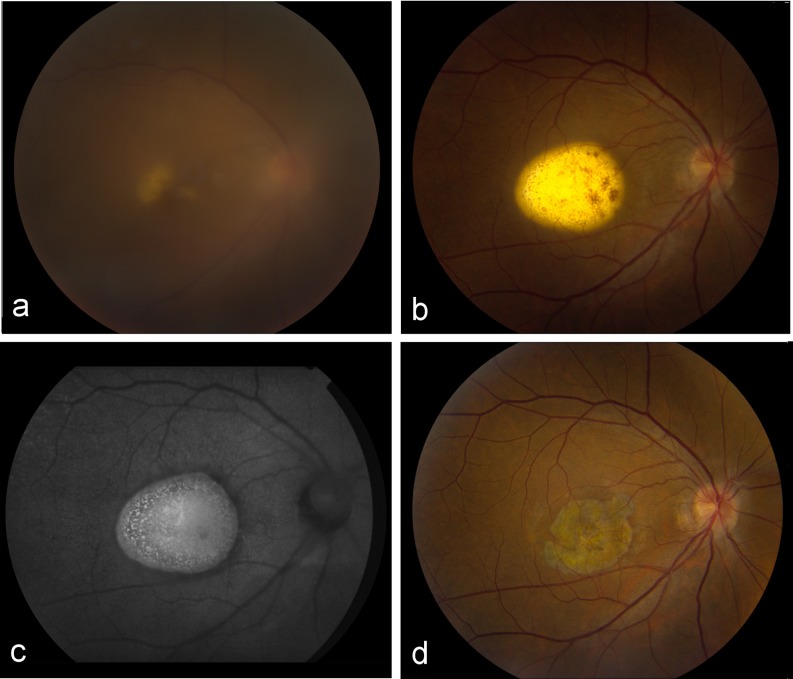
Fundoscopic images of the right eye of a 45-year-old female. **a** Uveitis and an underlying whitish, slightly elevated lesion. **b** Lesion after vitrectomy, 12 weeks later. **c** Autofluorescent image postvitrectomy. **d** Flat scar without signs of relapse, almost 2 years after chemotherapeutic treatment and irradiation

A 45-year-old female with a history of lupus nephritis class V presented with panuveitis of her right eye and loss of visual acuity of both eyes. During fundoscopy, a slightly elevated lesion was observed (Fig. 1a). As there was no systemic activity of her lupus erythematosus and the patient was immunocompromised due to chronic use of azathioprine and prednisolone, opportunistic infections were considered. No Herpes Simplex Virus, Varicella Zoster Virus, Cytomegalovirus, Epstein–Barr virus, toxoplasmosis, borreliosis, syphilis, or tuberculosis were found. Vitrectomy, however, revealed a rapidly growing lesion involving the macula lutea (Fig. 1b–c). Vitreous fluid analysis showed atypical lymphocytes and an interleukin-10 to interleukin-6 ratio of 14.8 (3,700 and 250 pg/mL, respectively), indicative of lymphoma rather than a non-neoplastic cause of uveitis. Brain magnetic resonance imaging, bone marrow, and spinal fluid analysis showed no abnormalities. Histology of the primary tumor could not be obtained as biopsy of this area would result in blindness. The patient was diagnosed with a solitary intraocular lymphoma and was treated with 2 cycles of intravenous methotrexate (3 g/m^2^, days 1 and 15); BCNU (100 mg/m^2^, day 4); teniposide (100 mg/m^2^, days 2–3); prednisolone (60 mg/m^2^, days 1–5); and intrathecal methotrexate, cytarabine, and hydrocortisone (15 mg, 40 mg, and 25 mg, respectively, days 1 and 15). The visual acuity of both eyes improved during treatment, suggesting a shrinking mass in both eyes. As there were no visible lesions in the left eye, only the right eye was irradiated (30 Gy). Six months later, the patient developed a lesion consistent with an intraocular lymphoma in the left eye, and at this time, radiotherapy was initiated for the left eye. Up-to-date, almost 2 years later, fundoscopy only shows flat scars, and the patient has no signs of ocular, cerebral, or systemic relapse (Fig. 1d).

Although high IL-10 levels are not pathognomonic for an intraocular lymphoma, patients with suspect lesions in regions which are essential for vision may benefit from vitreous interleukin measurement. If, in this way, a retinal biopsy can be avoided, eyesight can be retained. Furthermore, in patients presenting with a unilateral intraocular lymphoma, irradiation of the contralateral eye should be considered as up to 80 % actually have bilateral disease [3].
